# Plasma level of mannose-binding lectin is associated with the risk of recurrent pregnancy loss but not pregnancy outcome after the diagnosis

**DOI:** 10.1093/hropen/hoac024

**Published:** 2022-06-07

**Authors:** C Nørgaard-Pedersen, L H Rom, R Steffensen, U S Kesmodel, O B Christiansen

**Affiliations:** Department of Obstetrics and Gynaecology, Centre for Recurrent Pregnancy Loss of Western Denmark, Aalborg University Hospital, Aalborg, Denmark; Department of Clinical Medicine, Aalborg University, Aalborg, Denmark; Department of Obstetrics and Gynaecology, Centre for Recurrent Pregnancy Loss of Western Denmark, Aalborg University Hospital, Aalborg, Denmark; Department of Clinical Immunology, Aalborg University Hospital, Aalborg, Denmark; Department of Obstetrics and Gynaecology, Centre for Recurrent Pregnancy Loss of Western Denmark, Aalborg University Hospital, Aalborg, Denmark; Department of Clinical Medicine, Aalborg University, Aalborg, Denmark; Department of Obstetrics and Gynaecology, Centre for Recurrent Pregnancy Loss of Western Denmark, Aalborg University Hospital, Aalborg, Denmark; Department of Clinical Medicine, Aalborg University, Aalborg, Denmark

**Keywords:** recurrent pregnancy loss, habitual abortion, mannose-binding lectin, reproductive immunology, recurrent miscarriage

## Abstract

**STUDY QUESTION:**

Are low or high plasma mannose-binding lectin (p-MBL) levels associated with recurrent pregnancy loss (RPL) and the reproductive and perinatal outcomes before and after RPL?

**SUMMARY ANSWER:**

The prevalence of low p-MBL levels was significantly higher in RPL patients, while high levels were significantly less prevalent. No association was found between p-MBL level and reproductive and perinatal outcomes before and after RPL.

**WHAT IS KNOWN ALREADY:**

Mannose-binding lectin (MBL) is an important component in the innate immune system. Low p-MBL levels have been associated with RPL, while the correlation with high levels has been poorly studied. Adverse perinatal outcomes are generally more frequent among RPL patients, but reports concerning the association between maternal p-MBL levels and perinatal outcomes, including birth weight (BW) and gestational age (GA), are conflicting.

**STUDY DESIGN, SIZE, DURATION:**

This study was a combined cross-sectional and cohort study of 267 RPL patients admitted to the RPL Center of Western Denmark between January 2016 and March 2020. RPL patients were followed until birth of a liveborn child or until end of follow-up, March 2021. A sample of 185 healthy female blood donors of reproductive age was used as a MBL reference group.

**PARTICIPANTS/MATERIALS, SETTING, METHODS:**

All RPL patients had ≥3 consecutive pregnancy losses, a regular menstrual cycle and no known significant chromosomal or uterine malformations. At the first consultation, routine blood samples including p-MBL measurement and detailed obstetrical and perinatal information were collected. p-MBL levels in RPL patients were compared to the MBL reference group. A logistic regression analysis adjusted for relevant confounders assessed the association between low p-MBL levels and an unsuccessful reproductive outcome in RPL patients in first pregnancy after admission. Perinatal outcomes before and after RPL were compared between RPL subgroups according to low (≤500 µg/l), intermediate (501–3000 µg/l) and high (>3000 µg/l) p-MBL levels.

**MAIN RESULTS AND THE ROLE OF CHANCE:**

Significantly more RPL patients had low p-MBL levels (prevalence proportion ratio (PPR): 1.79, 95% CI: 1.34–2.38) and fewer had high p-MBL levels (PPR: 0.56, 95% CI: 0.40–0.79) compared to the reference group, while the prevalence of intermediate p-MBL level was not different between the groups (PPR: 0.86, 95% CI: 0.69–1.08). In the prospective study, low p-MBL level was not a significant risk factor for a pregnancy loss in the first pregnancy after admission after adjustment for age, BMI and smoking. Neither before nor after the RPL diagnosis were maternal p-MBL levels significantly associated with BW or GA.

**LIMITATIONS, REASONS FOR CAUTION:**

Only 161 (60.3%) patients had given birth after RPL during the follow-up period, which limited the possibility to detect clear associations between p-MBL levels and perinatal outcomes after RPL.

**WIDER IMPLICATIONS OF THE FINDINGS:**

In agreement with several previous studies, low p-MBL levels are strongly associated with RPL, while this study for the first time documents that high levels may play a protective role, which suggests a causal relationship. We suggest that larger prospective studies evaluate the association between p-MBL levels and RPL prognosis.

**STUDY FUNDING/COMPETING INTEREST(S):**

No external funding was received. We acknowledge the Department of Obstetrics and Gynaecology at Aalborg University Hospital for financial support. U.S.K. has reported personal fees from Merck, consulting fees from IBSA Nordic, and a grant from Gedeon Richter, Merck and IBSA Nordic outside of the submitted work.

**TRIAL REGISTRATION NUMBER:**

ID from clinicaltrials.gov is NCT04017754.

**WHAT DOES THIS MEAN FOR PATIENTS**? Mannose-binding lectin (MBL) is a protein in the blood that attaches to dead cells, viruses and bacteria and helps the immune system remove it from the circulation. The concentration of MBL in the blood is mainly determined by the genes inherited from one’s parents. A low concentration of MBL increases the risk of getting some infections, especially in newborns and HIV patients, and predisposes to some connective tissue diseases, while a high concentration seems to be harmful in other diseases. Previous studies have suggested that a low concentration of MBL is found more often in patients with recurrent pregnancy loss (RPL). However, we do not know if a low or high concentrations affect the pregnancy outcome before and after RPLs.

The present study concludes that a low MBL concentration is more frequent in patients with RPLs (defined as three or more consecutive pregnancy losses), but it does not have any clear impact on the chance of pregnancy and pregnancy outcome before and after the diagnosis, while a high MBL concentration seems to be protective against RPL. A low MBL concentration may be an indicator of an altered immune reaction to the foetus and placenta with a negative impact in first trimester but not throughout pregnancy and neither the foetus’ health at birth.

## Introduction

Early pregnancy loss occurs in about 15% of clinical pregnancies (Quenby *et al.*, 2021). Yet, 0.7–2.6% of women suffer from recurrent pregnancy loss (RPL) depending on what definition is used (Quenby *et al.*, 2021). The UK Royal College of Obstetricians and Gynaecologists and The German, Austrian and Swiss Society of Gynaecology and Obstetrics operate with the traditional definition of RPL defined as ≥3 consecutive miscarriages from the time of conception until 24 weeks of gestation (Rai *et al.*, 2011; Toth *et al.*, 2018). Other reproductive societies now define RPL as ≥2 miscarriages (Practice Committee of the American Society for Reproductive Medicine, 2020; The ESHRE Guideline Group on RPL *et al.*, 2018). Primary RPL (pRPL) is defined as RPL with no prior pregnancy carried to viability, whereas secondary RPL (sRPL) is defined as RPL after at least one pregnancy beyond 24 weeks of gestation (The ESHRE Early Pregnancy Guideline Development Group, 2019). Approximately 40% of RPL cases can be classified as sRPL (Jivraj *et al.*, 2001).

The prognosis of RPL worsens with increasing age, BMI and number of pregnancy losses, and also when the patient is exposed to tobacco smoking (Knudsen *et al.*, 1991; Metwally et al., 2010; The ESHRE Guideline Group on RPL *et al.*, 2018).

The aetiology of RPL often remains unknown; however, thrombophilia, endocrine disturbances, uterine anatomic abnormalities and chromosomal anomalies are risk factors that have been identified. Nevertheless, suggested risk factors can only be found in 20–50% of cases (Berger *et al.*, 2013). Much of the research in RPL risk factors has focused on a series of immunological disturbances with causative and prognostic impact on RPL. Biomarkers related to immunological dysfunction are found more often in RPL patients than expected (Kruse *et al.*, 2002; Christiansen *et al.*, 2006; King *et al.*, 2010; Ticconi *et al.*, 2010; Thangaratinam *et al.*, 2011; Wang *et al.*, 2016).

Mannose-binding lectin (MBL) is a C-type lectin promoting opsonization and phagocytosis of foreign antigens and activation of the complement system through the lectin pathway as part of the innate immune defence. Plasma MBL (p-MBL) levels fluctuate in early childhood, but in adult life, the p-MBL level is independent of age (Aittoniemi *et al.*, 1996; Ytting *et al.*, 2007; Sallenbach *et al.*, 2011; Gaya da Costa *et al.*, 2018). Some diseases are associated with low p-MBL levels, while other diseases are linked with high p-MBL levels (Bouwman *et al.*, 2006). Studies on RPL has focused primarily on low p-MBL levels without questioning the impact of high p-MBL levels. Kilpatrick *et al.* (1995) and Kruse *et al.* (2002) found low p-MBL levels in 15.6% and 18.9% of RPL patients, respectively, compared with <5.0% and 12.2% of controls.

MBL is an acute phase reactant and, therefore, the plasma levels change in response to factors such as pregnancy and infection (Kilpatrick, 2000; Herpers *et al.*, 2009). Nonetheless, the p-MBL level is mainly genetically determined by interactions of polymorphisms in the promoter and structural genes on chromosome 10, although other genetic factors also seem to be important (Swierzko *et al.*, 2009).

A significantly increased incidence of preterm birth, reduced birth weight (BW) and other perinatal complications is found in RPL patients (Basso *et al.*, 1998; Jivraj *et al.*, 2001; Brown *et al.*, 2008; Dempsey *et al.*, 2014; Field and Murphy, 2015; Rasmark Roepke *et al.*, 2021). Since low p-MBL levels may be associated with RPL, an obvious question is whether an aberrant p-MBL level is associated with a negative reproductive prognosis and an increased risk for adverse perinatal outcomes in RPL patients?

The aim of the present study was (i) to evaluate if the prevalence of RPL patients with low or high levels of p-MBL differs from the background population, (ii) if low p-MBL level is a risk factor for a pregnancy loss in the first pregnancy after admission and (iii) to evaluate whether low or high p-MBL levels are associated with negative reproductive and perinatal outcomes before and after RPL.

## Materials and methods

### Study population

From January 2016 to March 2020, 332 women admitted to The Center for Recurrent Pregnancy Loss of Western Denmark were identified in this combined cross-sectional and cohort study. Only patients with ≥3 consecutive spontaneous pregnancy losses were included. Both non-visualized and clinical losses (The ESHRE Early Pregnancy Guideline Development Group, 2019) documented in hospital records were acknowledged, whereas verified ectopic pregnancies, complete molar pregnancies and induced abortions for social and genetic reasons were not counted. The RPL patients were followed until birth of a liveborn child after RPL or until March 2021, whichever came first.

At the first consultation, all patients underwent a diagnostic work-up including collection of an obstetric and gynaecologic history, a routine blood analysis, a uterine hydrosonography or hysteroscopy and, in the majority of cases, also a chromosomal analysis of the RPL patient and her male partner. The p-MBL measurement has been included in the routine blood analysis since the RPL Center was established in 2016 because prior studies (Kilpatrick *et al.*, 1995; Christiansen *et al.*, 1999; Kruse *et al.*, 2002) listed in Table I suggested the biomarker to be associated with the condition.

**Table I:** Results from previous studies measuring plasma mannose-binding lectin level in recurrent pregnancy loss patients compared to controls.

Study	Ethnicity	p-MBL cut-off	RPL	%	Control	%	OR (*P*-value)
			N		N		
Kilpatrick *et al.* (1995)	Not described	<0.63 U/ml	21/135	15.6	23/280	8.2	1.89 (0.04)
Christiansen *et al.* (1999)	Danish and Scottish	<50 µg/l	30/195	15.4	41/444	9.2	1.67 (0.04)
Kruse *et al.* (2002)	Danish	<100 µg/l	41/217	18.9	38/314	12.2	1.46 (0.02)

p-MBL, plasma mannose-binding lectin; RPL, recurrent pregnancy loss.

According to the findings from the diagnostic work-up, treatment options were considered, including vaginal progesterone, oral prednisolone, oral levothyroxine, intravenous immunoglobulin (IVIG) infusion and/or subcutaneous heparin. In patients with an expected good prognosis, only psychological support and close monitoring (tender loving care) were offered.

Based on baseline characteristics, women were excluded if they had a significant uterine malformation (n = 2), age >45 years (n = 0), irregular and/or abnormal menstrual cycle length (<22 and >35 days interval) (n = 13), <3 consecutive pregnancy losses (n = 26) or no p-MBL measurement (n = 20). Also, if a significant chromosomal aberration was present in the RPL patient or her male partner, the patient was excluded except when donor semen or donor eggs had been used in relation to their former pregnancy losses (n = 4); we did not exclude RPL patients with positive tests for autoantibodies. In total, 267 RPL patients were included.

### MBL reference group

The MBL reference group comprised 185 Danish female, non-pregnant blood donors of reproductive age (range: 21 to 45 years), about whom we have no other information than their p-MBL level. After informed consent, the blood donors had an extra blood sample taken, when donating blood, which was analysed for the p-MBL level.

### Data collection

The protocol for the study was made public on clinicaltrials.gov (number NCT04017754) before data collection was completed. Relevant data were collected in all patients admitted to the RPL Center from their first consultation and until their contact to the clinic was terminated in the RPL clinical database of The RPL Center at Aalborg University Hospital, Denmark (Data Protection Agency of The North Denmark Region, Approval Number 2018-5). The data were stored in a Microsoft Access 2015 database.

Baseline characteristics collected at the first consultation included age, BMI, smoking habits, alcohol intake, number of stillbirths, live births and pregnancy losses. Data on perinatal outcomes of all births before and the first birth after RPL were collected from hospital records and, when needed, completed by telephone or e-mail correspondence. This included information about mode of delivery, gestational age (GA), BW, sex of the child, volume of peripartum haemorrhage, occurrence of preeclampsia and perinatal complications in pregnancy and during delivery.

Peripartum haemorrhage was routinely estimated and noted by the midwifes in the birth records generally by weighing absorbent bed sheets after vaginal delivery.

pRPL was defined as ≥3 consecutive pregnancy losses with no prior birth ≥22 weeks, while sRPL was defined as ≥3 consecutive pregnancy losses after ≥1 birth ≥22 weeks. In Denmark, 22 weeks of gestation is considered to be the cut-off for foetal viability. Live births refer to children born alive and who were still alive after 1 week. Stillbirth was defined as foetal death ≥22 weeks of gestation and with no signs of life after birth. Early and late miscarriage were defined as miscarriage prior to 12 weeks and between 12–21 + 6 weeks of gestation, respectively.

A negative reproductive outcome was defined as a pregnancy loss (clinical miscarriage or biochemical pregnancy) in the first pregnancy after admission in contrast to a positive reproductive outcome defined as a livebirth or an ongoing pregnancy >12 weeks at follow-up.

### Blood samples

The routine blood sample analysis was performed in all RPL patients in accordance with the recommendations from the ESHRE guideline on RPL (The ESHRE Guideline Group on RPL, 2018), and, in addition, the p-MBL level was measured. The list of autoantibodies tested for included anti-nuclear antibodies (ANAs), anti-double-stranded-DNA-antibodies and anti-thyroid peroxidase (TPO) antibodies, lupus anticoagulant, anti-cardiolipin antibodies (IgG/IgM ACAs) and anti-β_2_-glycoprotein-I antibodies (IgG/IgM a-β2-GPL-I). Blood samples were in 93.3% of the cases collected prior to pregnancy, and in 6.7% of cases taken in early pregnancy before gestational week 8.

### MBL assay

ELISA was used to measure p-MBL levels. Patient plasma was diluted 1:100 in dilution buffer (20 mM Tris + 10 mM CaCl_2_ + 1 M NaCl + 0.05% Triton X-100 + 0.1% BSA, pH 7.4) and incubated on mannan-coated ELISA microtitre wells (Mannose from Saccharomyces cerevisiae Sigma-Aldrich, Denmark, M-7504). After each step, wells were washed three times with washing buffer (Tris-buffered saline + 5 mM CaCl_2_ + 0.05% Tween 20) and incubated for a minimum of 1 h at room temperature. Biotin conjugated monoclonal anti-MBL (HYB 131-01B-0 1 mg/ml, from Antibody Shop, BioPorto Diagnostics) was added to the wells. Then, after incubation and washing, wells were incubated in alkaline phosphatase-conjugated Streptavidin (Dako code D0396, DakoCytomation, Denmark) and finally CSPD, a chemiluminescent phosphatase substrate, was added (ELISA-LIGHT CSPD/Sapphire-II T1023, Applied Biosystems, Life Technologies Europe BV, Denmark). After 20 min, the wells were analysed using a LUMIstar ELISA reader (BMG Labtech).

All quantifications were determined based on a standard calibration curve constructed with doubling dilutions of a highly purified MBL-standard (SER101). Each run was validated using three in-house control plasma samples from three different plasma donor patients with known p-MBL levels. If these in-house MBL controls were not within 2 SD of the standard curve, the whole run was rejected. The cut-off for low p-MBL used routinely in Danish laboratories is <500 µg/l. This method did not allow precise measurement of the exact plasma concentration of MBL when <100 µg/l and >5000 µg/l; thus, these levels were noted in the data collection as a plasma concentration of <100 µg/l and >5000 µg/l, respectively (Nørgaard-Pedersen and Christiansen, 2020, unpublished report).

### Statistical analysis

Data were analysed in Stata (MP 15.0 for Mac, revision 19, June 2017). A probability value <0.05 was considered significant. Patients were divided into three subgroups according to their p-MBL levels: low (≤500 µg/l), intermediate (501–3000 µg/l) and high p-MBL groups (>3000 µg/l). Baseline characteristics were listed for all RPL patients and for the three RPL subgroups according to p-MBL level. The latter subgroups were compared using the χ^2^ test for binary categorical variables. Normally distributed continuous variables were compared using unpaired one-way ANOVA and non-parametric variables using the Kruskal–Wallis test.

p-MBL levels were not normally distributed, and therefore median levels and 10th and 90th percentiles (P10/P90) were listed. Q–Q plots were used to assess the distribution of continuous variables.

Cross-sectional data on p-MBL levels were compared between RPL patients and the MBL reference group and analysed using the χ^2^ test; first comparing prevalence of ≤500 µg/l and >500 µg/l between the two groups and next the same procedure for intermediate and high levels, respectively. The relative prevalence of low MBL was compared using prevalence proportion ratio (PPR) with 95% CI.

Risk factor analysis within the RPL cohort was performed using a binominal logistic regression analysis. We tested if low p-MBL level (1 = p-MBL level ≤500 µg/l, 0 = p-MBL level >500 µg/l) in RPL patients was a risk factor for a negative reproductive outcome in the first pregnancy after admission (ongoing pregnancy >12 weeks and birth vs. new pregnancy loss). The analysis was adjusted for potential confounders measured at baseline including age (continuous), BMI (continuous) and exposure to tobacco smoke (1 = yes, 0 = no). The regression analysis was not adjusted for the number of consecutive pregnancy losses since p-MBL levels may have a causal relationship to the number of former losses, but a sensitivity analysis including this variable was performed. Results are presented as odds ratios (ORs) with 95% CI. Patients with no pregnancy after admission (N = 46), pregnant at first consultation (N = 18), and pregnant after admission but <12 weeks of gestation at final follow-up (N = 0) were not included in the logistic regression analysis of reproductive outcome.

Data on BW and GA were compared between RPL subgroups. Q–Q plots on continuous variables showed non-normal distribution and the three subgroups were therefore compared using the Kruskal–Wallis test for non-parametric variables whereas a two-sample Mann–Whitney *U* test was performed comparing the two RPL subgroups with p-MBL ≤500 µg/l and >500 µg/l, respectively. Between groups comparisons of the frequency of categorical variables were performed with a χ^2^ test or Fisher’s exact test when small numbers in one group was expected. Preterm birth was defined as birth <37 + 0 weeks, and low BW was defined as <2500 g. Peripartum haemorrhage included both peripartum haemorrhage during vaginal delivery and caesarean section A haemorrhage of 500–999 ml was considered moderate, whereas a haemorrhage ≥1000 ml was considered severe. Data on twins and stillbirths were not excluded from the analysis of reproductive outcome but were excluded from the analysis of GA and BW.

The test for linear association between low p-MBL levels (≤500 µg/l/>500 µg/l) and BW or GA of the first singleton livebirth before and after RPL was adjusted for the same potential confounders as the logistic regression analysing risk factors for reproductive outcome. In addition, the linear regression analysis for the association between low p-MBL levels and BW was adjusted for GA (continuous).

### Sex of children born before RPL

When analysing sex of previous births, only sRPL patients with a previous birth >22 weeks of gestation of one child or several children of the same sex were included (N = 108). The 15 sRPL patients with previous birth of children of mixed sexes were excluded from this analysis. Sex ratio was reported as a male:female ratio.

### Ethical approval

The database was approved by The Danish Data Protection Agency of The North Denmark Region (Approval Number 2018-5). Since only data on routine investigations and interventions in the RPL Center were analysed and reported, no permission from the ethics committee was required.

## Results

### Patient characteristics

In the 267 RPL patients included, baseline characteristics did not differ between patients with p-MBL levels ≤500 µg/l and >500 µg/l (Table II).

**Table II:** The baseline characteristics of all recurrent pregnancy loss patients.

Characteristics	All RPL patients	≤500 μg/l	501–3000 μg/l	>3000 µg/l	*P* ^a^
		(N = 119)	(N = 102)	(N = 46)	
**Age, years, mean (** ± **SD)**	33.2 (±5.3)	33.4 (±5.3)	32.8 (±5.5)	33.6 (±5.1)	0.84
**BMI,** ^b^ **kg/m^2^, median (P10/P90)**	25.4 (20.0/34.5)	25.5 (19.5/34.0)	25.0 (20.0/35.0)	26.5 (20.5/33.5)	0.20
**Maternal smoking, N (%)**	35 (13.1)	16 (13.4)	13 (12.7)	6 (13.0)	1.0
**No. of consecutive pregnancy losses, median, N (range)**	3 (3–13)	3 (3–13)	3 (3–8)	4 (3–10)	0.87
**pRPL, N (%)**	144 (53.9)	65 (54.6)	59 (57.8)	20 (43.5)	0.26
**sRPL, N (%)**	123 (46.1)	54 (45.4)	43 (42.2)	26 (56.5)	
**Treatment after admission, N (%)**					
** IVIG**	85 (31.8)	40 (33.6)	30 (29.4)	15 (32.6)	0.79
** ART**	78 (29.2)	37 (31.1)	30 (29.4)	11 (23.9)	0.66

RPL, recurrent pregnancy loss; pRPL, primary RPL; sRPL, secondary RPL; IVIG, intravenous immunoglobulin; ART, IVF and ICSI; p-MBL, plasma mannose-binding lectin.

a *P*-value for differences between the three RPL subgroups according to p-MBL level.

b Two missing values.

### p-MBL level

RPL patients had a mean age of 33 years (SD ±5.3) and a median p-MBL level of 717 µg/l (P10/P90: 100/4069 µg/l), while age and p-MBL levels in the MBL reference group were significantly higher with a mean age of 35.2 (SD ±5.7) years (*P* < 0.001) and a median p-MBL level of 1717 µg/l (P10/P90: 100/5000 µg/l). Eighteen (6.7%) patients were pregnant <12 weeks of gestation at their first consultation when the blood sample was collected, whereas no participants in the MBL reference group were pregnant. The median p-MBL level measured in early pregnancy was 218 µg/l (P10/P90: 100/3307 µg/l), and it was significantly lower than the mean p-MBL level of 801 µg/l (P10/P90: 100/4134 µg/l) measured in blood samples taken before pregnancy. Fourteen (77.8%) of the 18 pregnant patients had a p-MBL level ≤500 µg/l compared to 105 (42.2%) of 249 non-pregnant patients (*P* = 0.003).

**Figure 1.**
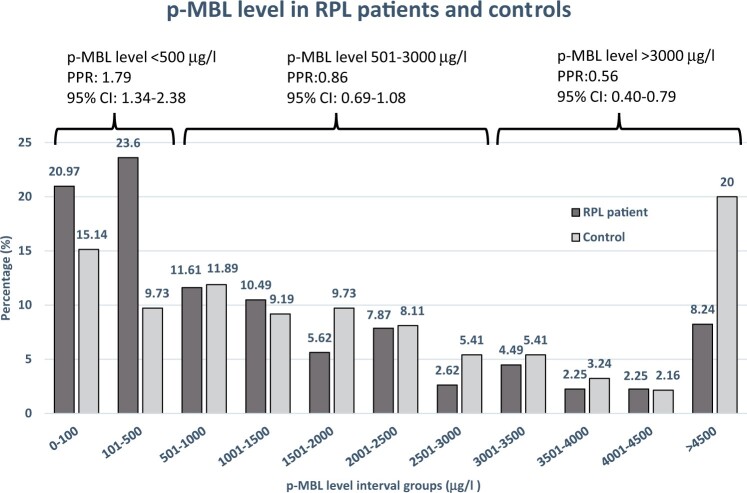
**Plasma mannose-binding lectin (p-MBL) levels in primary recurrent pregnancy loss (pRPL) patients and the mannose-binding lectin (MBL) reference group.** Percentage in the RPL group and the reference group with plasma p-MBL levels within each p-MBL level interval group is listed above each bar. The prevalence proportion ratios (PPRs) and 95% CI for comparison of prevalence of RPL patients (N = 267) and controls (N = 185) having a low (≤500 µg/l), intermediate (501–3000 µg/l) and high (>3000 µg/l) p-MBL level are shown above each curly bracket.

We observed a significantly higher prevalence of p-MBL level ≤500 µg/l in all RPL patients compared to the MBL reference group (44.6% vs. 24.9%; PPR: 1.79, 95% CI: 1.34–2.38) (Fig. 1). When patients who were pregnant at the first consultation were excluded from this analysis, the difference remained significant (42.2% vs. 24.9% PPR: 1.70, 95% CI: 1.27–2.27). With a p-MBL cut-off level ≤100 µg/l, the prevalence in RPL patients compared to the MBL reference group did not differ significantly (21.0% vs. 15.1%, PPR: 1.39, 95% CI: 0.92–2.09). In contrast, a p-MBL level >3000 µg/l was significantly less prevalent among RPL patients compared with the MBL reference group (17.2% vs. 30.8%, PPR: 0.56, 95% CI: 0.40–0.79). The prevalence of p-MBL levels ≤500 µg/l did not differ between RPL patients with 3 consecutive pregnancy losses compared to patients with ≥4 consecutive pregnancy losses (44.9% vs. 44.1%, PPR: 0.98, 95% CI: 0.75–1.29).

### The impact of p-MBL level on reproductive outcome

**Figure 2.**
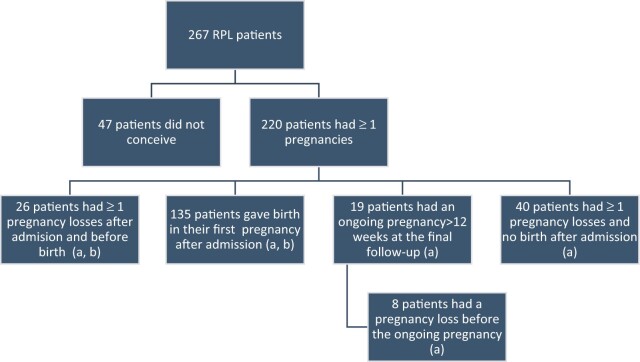
**The distribution of the accumulated reproductive outcome of all recurrent pregnancy loss (RPL) patients.** Boxes marked a: These patients are included in the analysis of the effect of low plasma mannose-binding lectin (p-MBL) level on reproductive outcome after RPL. Boxes marked b: Perinatal outcomes from these patients’ births are included in the analysis of the effect of low p-MBL level on perinatal outcome after RPL.

In a cumulative analysis of reproductive outcome after admission and before final follow-up, 161 (60.3%) RPL patients had given birth to a liveborn child, and 19 (7.1%) RPL patients were pregnant beyond 12 weeks of gestation (ongoing pregnancy) (Fig. 2). Considering only the first pregnancy outcome after admission, 135 RPL patients had a livebirth, and 11 RPL patients had an ongoing pregnancy at final follow-up. Four RPL patients had twins whereas no stillbirths were observed after RPL.

The adjusted impact of low p-MBL levels on the reproductive outcome in the first pregnancy after admission was not significant (Table III). Increasing age and BMI were significant risk factors for a negative reproductive outcome.

**Table III:** Logistic regression analysis of the association between low plasma mannose-binding lectin levels and a pregnancy loss in first pregnancy after admission, in all recurrent pregnancy loss patients (N = 200), adjusted for relevant confounders.

All RPL	OR (95% CI)	*P*
**p-MBL ≤500 μg/l**	0.61 (0.33–1.15)	0.13
**Age^a^**	1.07 (1.01–1.14)	0.02
**BMI** ^**b**^ ^**,c**^	1.06 (1.01–1.12)	0.03
**Smoking**	2.42 (0.92–6.33)	0.07

RPL, recurrent pregnancy loss; p-MBL, plasma mannose-binding lectin; OR, odds ratio.

a Per year.

b Per BMI unit.

c Two women had missing BMI values.

A sensitivity analysis (not shown) adjusting for the number of consecutive pregnancy losses at the time of admission yielded ORs for low p-MBL levels and the other competing risk factors of comparable magnitude to those in Table III.

### Perinatal outcome

The BW and GA in live births and the frequency of a moderate and severe peripartum haemorrhage did not depend on p-MBL levels, either before or after the RPL diagnosis (Tables IV–VI). According to perinatal outcomes in the RPL patients with very low p-MBL <100 µg/l, mean GA of the first born child before and after admission was 279 (±12) days and 277 (±11) days, respectively, and BW was 3345 (±604) g and 3446 (±419) g, respectively. These perinatal outcomes did not differ significantly from the outcomes observed in the three RPL subgroups listed in Tables III and IV.

**Table IV:** Perinatal data of first birth ≥22 weeks before sRPL according to plasma mannose-binding lectin levels.

p-MBL	≤500 μg/l	501–3000 μg/l	>3000 μg/l	All sRPL
	(N = 54)	(N = 43)	(N = 26)	(N = 123)
**BW,^a^ g, mean (** ± **SD)**	3429 (±614)	3385 (±554)	3421 (±461)	3412 (±560)
**Low BW, N (%)** **(N = 114)**	4 (7.8)	2 (5.3)	2 (8.0)	8 (7.0)
**GA,^a^ days, mean (** ±SD)	278 (±13)	278 (±13)	276.3 (±9)	278 (±12)
**Preterm birth, N (%)** **(N = 109)**	4 (8.2)	4 (10.8)	1 (4.4)	9 (8.3)
**Sex,** ^**b**^ **N (%) of boys **(N = 108)****	36 (76.6)^c^	21 (53.9)	15 (65.2)	72 (66.1)
**Peripartum haemorrhage**				
≥ **500 ml, N (%)**	14 (31.1)	10 (26.3)	6 (28.6)	30 (29.8)
≥ **1000 ml, N (%)**	8 (17.8)	6 (15.8)	0 (0.0)	14 (13.5)
**(N = 104)**				

BW, birth weight; GA, gestational age; sRPL, secondary recurrent pregnancy loss; p-MBL, plasma mannose-binding lectin; OR, odds ratio.

a Data on one set of twins and eight stillbirths were not included in the analysis of gestational age and birth weight.

b Excluded from analysis: 15 women with children of both sexes of previous births.

c OR 2.36 95% CI: 1.02–5.49 comparing p-MBL level ≤500 µg/l to p-MBL levels >500 µg/l.

**Table V:** Perinatal data of first birth ≥22 weeks after recurrent pregnancy loss according to plasma mannose-binding lectin levels.

p-MBL	≤500 μg/l	501–3000 μg/l	>3000 μg/l	All RPL
	(N = 80)	(N= 62)	(N = 23)	(N = 165)
**BW,^a^ g, mean (** ± **SD)**	3467 (±633)	3389 (±650)	3372 (±578)	3425 (±530)
**Low BW, N (%)** **(N = 152)**	3 (4.0)	5 (9.1)	1 (4.8)	9 (5.9)
**GA,^a^ days, mean (** ± **SD)**	274 (±21)	275 (±16)	274 (±13)	274 (±18)
**Preterm birth, N (%)** **(N = 157)**	8 (10.3)	8 (14.0)	3 (15.8)	19 (12.1)
**Sex, N (%) of boys (N = 160)**	41 (51.9)	30 (51.7)	16 (69.6)	87 (54.4)
**Peripartum haemorrhage**				
≥ **500 ml, N (%)**	27 (34.6)	21 (36.2)	5 (21.7)	53 (33.3)
≥ **1000 ml, N (%)**	6 (7.7)	8 (13.8)	3 (13.0)	17 (10.7)
**(N = 159)**				

p-MBL, plasma mannose-binding lectin; RPL, recurrent pregnancy loss; BW, birth weight; GA, gestational age.

a Data on four set of twins were not included in the analysis of gestational age and birth weight.

Missing data: nine BW values, four GA values and six peripartum haemorrhage values. Three twin births were of same sex, while one set of twins was of different sexes; thus, one set of twins and four missing sex values was not included in the analysis.

**Table VI:** Linear regression coefficients for the impact of a low (≤500 μg/l) compared to a higher (>500 μg/l) plasma mannose-binding lectin level on gestational age and birth weight of the firstborn child before and after the recurrent pregnancy loss diagnosis.

	Firstborn before RPL	Firstborn after RPL
**LRC for BW, g (95% CI)**	108 (−67 to +282)	22 (−147 to +191)
**LRC for GA, days (95% CI)**	−1 (−7 to +5)	1 (−4 to +6)

RPL, recurrent pregnancy loss; LRC, linear regression coefficients; BW, birth weight; GA, gestational age.

Among sRPL patients with p-MBL levels ≤500 µg/l, 76.6% had previously given birth to a boy(s) only, which differed significantly from the 58.1% of sRPL patients with p-MBL levels >500 µg/l (OR 2.36 95% CI: 1.02–5.49) (Table IV). This corresponds to a sex ratio of 3.3 in births before sRPL among patients with p-MBL ≤500 µg/l, whereas among sRPL patients with p-MBL >500 µg/l the sex ratio was 1.3. After RPL, the sex ratio was 1.1 among RPL patients with p-MBL ≤500 µg/l and 1.3 among RPL patients with p-MBL >500 µg/l.

The adjusted linear regression analysis showed no significant association between p-MBL level and GA or BW, either before or after RPL since all of the linear regression coefficients had 95% CIs including 0 (Table VI).

The frequency of severe peripartum haemorrrhage did not differ significantly between patients with p-MBL ≤ and >500 µg/l (Table IV). No patients with a p-MBL level >3000 µg/l had a severe peripartum haemorrhage before RPL. When taking all previous births into account, 9 (19.6%) patients with low p-MBL, 7 (17.1%) patients with intermediate p-MBL levels and 0 patients with a high p-MBL level had at least had one severe peripartum haemorrhage in one of the deliveries before sRPL.

### Autoantibodies

In the present study, 36.2%, 38.2% and 28.3% RPL patients with p-MBL levels ≤500 µg/l, p-MBL 501–3000 µg/l and p-MBL >3000 µg/l, respectively, were positive for one or more of the autoantibodies investigated routinely in our RPL Center. There were no significant associations between any of the three subgroups based on p-MBL level and the presence of any of the investigated antibodies. Nonetheless, 11.0% of patients with p-MBL levels ≤500 µg/l and 18.6% of patients with p-MBL levels 501–3000 µg/l had antibodies against TPO in contrast to only 2.2% with p-MBL levels >3000 µg/l (PPR: 0.14 95% CI 0.02–1.07).

## Discussion

### p-MBL levels and RPL

The prevalence of a low p-MBL level (≤500 µg/l) was 1.79 times higher among RPL patients compared to the MBL reference group, whereas the prevalence of a high p-MBL level (>3000 µg/l) was significantly less frequent among RPL patients.

These results partly agree with the results of previous studies evaluating p-MBL levels in relation to RPL; yet these studies have not come up with a mutual agreement on the cut-off value for a low p-MBL level. One prior study found an increased frequency of low p-MBL levels defined as ≤50 µg/l or ≤100 µg/l in RPL patients compared to controls, whereas a cut-off value of 200 µg/l appeared to reduce the observed association (Kruse *et al.*, 2002). In this study, the frequency of low p-MBL levels also increased with the number of previous pregnancy losses. In contrast, the present study did not observe a positive correlation between the prevalence of low p-MBL levels and the number of pregnancy losses at the time of admission and also, with a p-MBL cut-off value of 100 µg/l, the prevalence between RPL patients and the reference group did not differ significantly.

From a physiological and clinical point of view, the optimal cut-off value for low p-MBL levels still has to be determined: either across diseases or solely concerning RPL patients. In Danish laboratories, a p-MBL level ≤500 µg/l is defined as insufficient, and this is in agreement with the cut-off value used in previous studies investigating the impact of p-MBL levels in diseases such as severe acute respiratory syndrome coronavirus infection, chronic obstructive lung disease, systemic lupus erythematosus (SLE) and lupus nephritis (Ip *et al.*, 2005; Eagan *et al.*, 2010; Albert *et al.*, 2012; Perazzio *et al.*, 2016). The optimal cut-off value probably varies between diseases but may also depend on the applied method of measurement. The assay for measuring p-MBL levels used in our laboratory today differs from the assays used in the previous studies (Kilpatrick *et al.*, 1995; Christiansen *et al.*, 1999; Kruse *et al.*, 2002), which may be partly responsible for the altered distribution of low p-MBL levels seen over the years.

While the prevalence distributions in patients and controls indicated that an intermediate p-MBL level was neutral with regard to RPL risk, a high p-MBL level seemed to protect against RPL. This finding demonstrates a biological gradient between p-MBL levels and RPL, which has not been reported in previous studies, and it strengthens our hypothesis that a low p-MBL level is indeed causally related to the risk of RPL.

The mean age in the MBL reference group was significantly higher than in RPL patients; however, we consider this small difference of no significance since previous studies have shown that there is only a weak (and possibly not clinically relevant) negative correlation (Troldborg *et al*., 2017) or no association at all between p-MBL levels and age in adults (Aittoniemi *et al.*, 1996; Ytting *et al.*, 2007; Sallenbach *et al.*, 2011; Gaya da Costa *et al.*, 2018). We are aware that we cannot prove that the p-MBL levels, measured after the patient had experienced ≥3 pregnancy losses, had not changed from the level before these events. Nonetheless, since the p-MBL level is mainly genetically determined and does not depend on age, we have no reason to believe that RPL patients with low p-MBL levels at the time of admission did not have low levels before their RPLs as well. Our findings therefore support our hypothesis that a low p-MBL level is a risk factor for RPL.

Since p-MBL levels are partially determined by polymorphisms in the MBL-2 gene, some studies in RPL patients have been based on MBL-2 genotyping rather than measurement of plasma concentration of the MBL protein. Two previous studies deducing MBL deficiency from MBL-2 genotyping found no evidence of MBL deficiency being a risk factor for RPL (Baxter *et al.*, 2001; Berger *et al.*, 2013). In a small cross-sectional study, Baxter *et al.* (2001) found a small but insignificant difference in the prevalence of low MBL-associated MBL-2 genotypes between 76 RPL patients with ≥3 miscarriages and 69 female controls. Berger *et al.* (2013), who made a similar comparison between 219 women with ≥2 not necessarily consecutive miscarriages and 236 female controls, found no significant association between low-producing MBL-2 genotype and RPL. Yet, it is noteworthy that Berger *et al.* (2013) found a remarkably lower frequency of low-producing MBL-2 genotypes compared to other studies on frequencies of MBL-2 genotypes in Caucasians (Kruse *et al.*, 2002; Christiansen *et al.*, 2009). Such a difference may be due to different genetic compositions of the investigated populations or be caused by inaccuracy of the genotyping techniques in the study by Berger *et al.* (2013).

The conflicting results between studies deducing p-MBL levels from MBL-2 genotyping (Baxter *et al.*, 2001; Berger *et al.*, 2013) and studies measuring the level directly in plasma (Kilpatrick *et al.*, 1995; Kruse *et al.*, 2002; Christiansen *et al.*, 2009) can be due to a limited reliability of the former method to predict the p-MBL level. Garred *et al* (2003), Steffensen *et al.* (2000), Swierzko *et al.* (2009) and Wang *et al.* (2016) observed a 10- to 56-fold variation in p-MBL levels within some of the MBL-2 genotype groups, and therefore concluded that inferring p-MBL levels solely from MBL-2 genotypes is inaccurate for individuals. Although monozygotic twin studies suggest that a p-MBL level is highly genetically determined (Sorensen *et al.*, 2007), only a proportion of this variability lays in the recognized MBL-2 genetic polymorphism. Possibly, we still do not know all the polymorphic sites that determine the MBL level.

Based on the findings of a higher prevalence of low p-MBL levels in RPL patients and the known action of MBL in phagocytosis and clearance of apoptotic and necrotic cells (Nauta *et al.*, 2003), we speculate that MBL deficiency causes a slower and/or insufficient clearance of the excessive number of apoptotic and necrotic placental and foetal cells passing into the maternal circulation during a pregnancy loss or delivery which in turn causes a high level of cells with alloantigens activating the woman’s immune cells (Stuart *et al.*, 2005; Minas *et al.*, 2007). This state is associated with an increased risk of autoimmune disease (Clemens *et al.*, 2000; Stuart *et al.*, 2005; Darrah and Andrade, 2013). The activated immune cells may have B- or T-cell receptors specific for the alloantigens on the foetal, trophoblast or syncytiotrophoblast cells. Thus, in a new pregnancy, these immune cells are exposed to the same antigens, which consequently may initiate a harmful cellular immune response increasing the risk of pregnancy loss or pregnancy complications similar to the Rhesus D immunization (Minas *et al.*, 2007; Tiblad *et al.*, 2013; Atia, 2017). If such immunization is involved in the pathogenesis of some cases of RPL, these women may benefit from immunomodulatory treatment bringing their immune defense under control.

### Reproductive outcome after RPL

A low p-MBL level was not a significant risk factor for a negative reproductive outcome in a first pregnancy after admission when adjusted for relevant confounders. The ORs listed on the confounding variables support our expectation that these factors indeed did influence significantly the reproductive outcome which is in accordance with the well-established literature on the impact of increasing age, BMI and smoking on pregnancy prognosis. This indicates that our cohort of RPL patients was representative of the RPL population and strengthens the credibility and validity of the results in the present study.

A previous Danish prospective study showed that RPL patients with p-MBL ≤100 µg/l had a 31.0% lower chance of live birth in their first subsequent pregnancy compared to RPL patients with higher p-MBL levels; a reduction that was statistically significant, but was not adjusted for confounders (Kruse *et al.*, 2002). Certainly, the number of previous pregnancy losses could explain these opposing results. The frequency of low p-MBL levels did indeed increase with increasing number of previous pregnancy losses in the study by Kruse *et al.* (2002) in contrast to our study. Increasing number of previous miscarriages is a well-defined negative prognostic factor for the pregnancy outcome (Clifford *et al.*, 1997; Li *et al.*, 2002; Lund *et al.*, 2012). In our study, we speculated that increasing number of pregnancy losses was to some degree a consequence of low p-MBL levels rather than other MBL-independent covariates. Therefore, we performed the regression analysis without adjustment for the number of previous pregnancy losses, but we also supplemented with a sensitivity analysis including the adjustment. In the sensitivity analysis, the number of pregnancy losses at admission was not a significant confounder, and a low p-MBL level did not have negative prognostic impact on pregnancy outcome. It is possible that the more intensive immunomodulatory treatment with IVIG, which was offered to 31.8% of patients in the present study, tended to cancel out the negative effect on reproductive outcome found in the previous study undertaken from 1988 to 1999 where approximately only 17% of patients were offered IVIG (Kruse *et al.*, 2002). Also, treatments with prednisolone and vaginal progesterone are offered much more frequently now.

### Birth weight and gestational ages

In the present study, we found no association between p-MBL levels and GA or BW, neither before nor after RPL in the non-adjusted and adjusted analysis. Also, we found no association between low p-MBL levels and the incidence of preterm birth and low BW, although the latter analyses were based on very few observations. Nonetheless, the frequency of preterm birth after RPL in our patients corresponds to the frequency of 10.0% reported in a large Swedish register study (Rasmark Roepke *et al.*, 2021).

In the Danish prospective study previously described, the median BW of the firstborn child born at term of RPL patients with a low p-MBL level (≤100 µg/l) was 287 g lower compared to that of patients with a normal p-MBL level in a non-adjusted analysis, whereas the prevalence of preterm birth (<37 weeks) was not associated with a low p-MBL level (Kruse *et al.*, 2002). In non-RPL women, another study reported an association between preterm birth and a reduced p-MBL level (Koucký *et al.*, 2018).

In non-RPL women, one study reported that a low MBL-producing MBL-2 genotype was associated with preterm birth (<29 weeks of gestation) (Annells *et al.*, 2004), whereas another study found no association between preterm birth and MBL2-genotypes (Wang *et al.* 2016). In contrast, in non-RPL women, one study found an association between high MBL-producing MBL-2 genotypes and an increased incidence of preterm birth (<37 weeks of gestation) compared with nulliparous women with intermediate and low MBL-producing genotypes (van de Geijn *et al.*, 2008).

These conflicting and inconclusive results from studies of MBL in relation to reduced BW and preterm birth call for more and larger studies on this topic.

### Sex of firstborn child and RPL

In sRPL patients with p-MBL levels ≤500 µg/l, the frequency of RPL patients with boy(s) only in the birth(s) before RPL was significantly higher (76.6%) than in patients with p-MBL levels >500 µg/l (58.7%, *P* = 0.05) corresponding to a sex ratio 3.3 and 1.3, respectively. Since there is no rationale for suggesting that low p-MBL levels can affect sex ratio (e.g. increased survival of male embryos), the most plausible explanation for the very high sex ratio in sRPL patients with low p-MBL levels is that the combination of a low maternal p-MBL level and a firstborn boy predisposes to sRPL more strongly than a firstborn boy alone. However, more studies are needed to clarify this association.

### Severe peripartum haemorrhage and RPL

In the sample of sRPL patients, we found an unexpectedly high prevalence of severe peripartum haemorrhage before RPL. It is noteworthy that none of these patients with a history of a severe peripartum haemorrhage had a high p-MBL level, whereas 17.8% and 15.8% of patients with low and intermediate p-MBL levels did. In births after RPL, the prevalence of severe peripartum haemorrhage was more equally distributed between subgroups. We speculated that a severe peripartum haemorrhage is an indicator of an increased transfer of trophoblast and foetal cells into the maternal circulation, which increases the risk of maternal immunization against foetal alloantigens, especially if the level of p-MBL is not abundant enough to help quickly eliminate and clear the maternal circulation from these cells. Such immunization may predispose to subsequent RPL. Since the finding of a high prevalence of severe perinatal haemorrhage before RPL was a novel and unexpected finding, it might be a chance finding, which must be repeated in a new and larger patient group before firm conclusions about its role in the pathogenesis of sRPL can be made.

### Low p-MBL and autoimmunity

Low MBL-producing genotypes have been associated with susceptibility to and severity of autoimmune diseases such as rheumatoid arthritis and SLE (Saevarsdottir *et al.*, 2001; Garred *et al.* 2001). Although higher prevalence of low p-MBL levels was found in RPL patients and both a low p-MBL level and RPL are associated with autoimmune disorders (Wang *et al.* 2016), the presence of autoantibodies in RPL patients was not in general associated with MBL deficiency. Nonetheless, TPO antibodies were elevated in significantly fewer RPL patients with high MBL levels (>3000 µg/l) compared to patients with lower MBL levels. Further studies must be undertaken on the topic.

### Limitations of the study

The present study focused solely on p-MBL levels; however, as prior studies have found no association between specific MBL-2 genotypes and RPL, and the association between known MBL-2 genotypes and plasma level can vary abundantly, we do not expect that genotyping our cohort would add new evidence. Also, the study measured p-MBL levels using mannan-binding ELISA: this method is preferred over the double-antibody ELISA since it measures p-MBL multimers and not the non-functional monomers, dimers, and trimers and the level is therefore a direct indication of the MBL ligand-binding or opsonization capability. The p-MBL levels measured with mannan-binding ELISA also have the strongest correlation with the complement-activating capacity (Minchinton *et al.*, 2002). Therefore, we consider our data on p-MBL levels as more precise when searching for associations between MBL and RPL compared to other analysis methods. However, the number of pregnancies after referral was limited, and the conclusions about the impact of p-MBL levels on subsequent reproductive and perinatal outcome must be interpreted with care.

Since MBL is an acute phase reactant, a p-MBL level depends to some degree on factors such as infection and pregnancy, which may in theory confound the results. During pregnancy, small insignificant changes have been reported in patients with low MBL-producing MBL-2 genotypes, whereas moderate increases of p-MBL have been reported in patients with MBL-2 alleles associated with normal p-MBL levels (Kilpatrick, 2000; van de Geijn *et al.*, 2007). Nevertheless, in our study, median p-MBL levels measured in early pregnancy were significantly lower compared to non-pregnant RPL patients (*P* = 0.020), but even after exclusion of the 18 RPL patients who were pregnant when blood samples were taken, the prevalence of p-MBL level ≤500 µg/l remained significantly increased in RPL patients compared with controls. Since data on p-MBL during pregnancy are at odds with previous studies, further studies on this topic are needed.

## Conclusions

Significantly more RPL patients have a low p-MBL level and less RPL patients have a high p-MBL level compared to fertile female blood donors, whereas an intermediate p-MBL level appeared with equal prevalence in RPL patients and controls. This biological gradient together with the analogy and the consistency seen in among previous studies on MBL and RPL support the theory that MBL has a contributory causal relationship with RPL. However, p-MBL levels do not seem to play an important role for the subsequent reproductive and perinatal outcome. As a new finding in patients with sRPL, a previous severe peripartum haemorrhage and/or the previous birth of a boy may interact in the pathophysiology of sRPL. This new observation must be confirmed in further observational studies in sRPL patients and in laboratory studies which could aim at demonstrating an association between p-MBL levels in the maternal plasma and clearance rate of foetal/trophoblast cells from maternal blood after delivery.

## Data availability

The data underlying this article will be shared on reasonable request to the corresponding author.
